# Jasmonates Promote β-Amylase-Mediated Starch Degradation to Confer Cold Tolerance in Tomato Plants

**DOI:** 10.3390/plants13081055

**Published:** 2024-04-09

**Authors:** Xiulan Fan, Huanru Lin, Fei Ding, Meiling Wang

**Affiliations:** School of Life Sciences, Liaocheng University, Liaocheng 252000, China; 17861828160@163.com (X.F.); linhuanru6740@163.com (H.L.)

**Keywords:** JA, MYC2, β-amylase, cold stress, tomato

## Abstract

Cold stress severely restricts growth and development, reduces yields, and impairs quality in tomatoes (*Solanum lycopersicum*). Amylase-associated starch degradation and soluble sugar accumulation have been implicated in adaptation and resistance to abiotic stress. Here, we report a β-amylase (BAM) gene, *SlBAM3*, which plays a central role in tomato cold tolerance. The expression of *SlBAM3* was triggered by cold stress. *SlBAM3* knockout using the CRISPR/Cas9 system retarded starch degradation and reduced soluble sugar accumulation in tomato plants, eventually attenuating cold tolerance. Expression analysis revealed that the *SlBAM3* transcript level was boosted by MeJA. Furthermore, MYC2, an essential component of the JA signaling pathway, could bind to the *SlBAM3* promoter and directly activate *SlBAM3* transcription, as revealed by yeast one-hybrid and dual LUC assays. In addition, the suppression of *MYC2* resulted in increased starch accumulation, decreased soluble sugar content, and reduced tolerance to cold stress in tomato plants. Taken together, these findings demonstrate that JA positively regulates β-amylase-associated starch degradation through the MYC2-SlBAM3 module in tomato during cold stress. The results of the present work expand our understanding of the mechanisms underlying *BAM* gene activation and starch catabolism under cold stress. The regulatory module of *SlBAM3* can be further utilized to breed tomato cultivars with enhanced cold tolerance.

## 1. Introduction

Plants are constantly challenged by various abiotic stress factors, among which cold is considered the most serious, especially in the current scenario where the frequency and magnitude of extreme weather events are increasing [1,2,3]. Cold stress impairs plant growth and development, and reduces crop productivity. It has been well established that low temperatures abate photosynthesis, disrupt membrane integrity, inactivate enzymes, and generate excessive reactive oxygen species [2,4,5,6,7]. Being sessile in nature, plants have evolved a broad range of strategies involving physiological, molecular, biochemical, and metabolic adjustments to survive under cold conditions. A favorable strategy for plants to cope with cold stress is to synthesize and accumulate a diversity of compatible solutes, also known as osmolytes, including soluble sugars, proline, and betaine [8,9,10]. Soluble sugars play a crucial role in tolerance to low temperatures in plants. They function mainly by preventing or alleviating membrane damage, protein inactivation, and excessive ROS accumulation [11,12,13].

Soluble sugars include sucrose, glucose, fructose, and maltose, among others. A series of enzymes are involved in the biosynthesis and catabolism of sugars, among which β-amylase (BAM) is essential for the degradation of transitory starch and the subsequent production of maltose, glucose, and sucrose [14,15]. BAM functions as an exoamylase that hydrolyzes the α-1,4 glycosidic bonds of starch, leading to the generation of maltose. Maltose is then transported to the cytosol and serves as a substrate to be metabolized for the generation of glucose and/or sucrose [15,16]. BAM genes have been identified in several plant species, with those in Arabidopsis being well characterized. There are a total of nine *BAM* genes in Arabidopsis, of which *BAM1/2/3* encodes catalytically active BAMs known to be localized in the chloroplast, where they directly catalyze the degradation of starch [17,18]. It is notable that the functions of BAMs are not limited to starch metabolism. An increasing number of studies point to the critical function of *BAM* during environmental stress in plants. For instance, *PtrBAM3* is activated by drought in trifoliate orange (*Poncirus trifoliata*), and its ectopic expression in lemon tree accelerates starch degradation and promotes drought tolerance [19]. Similarly, olive (*Olea europaea*) and apple (*Malus domestica*) *BAM* genes are substantially up-regulated under water-deficient conditions [20,21]. Additionally, *IbBAM1.1* regulates ROS homeostasis and confers drought and salt tolerance in sweet potato (*Ipomoea batatas*) [22]. *BAM* genes have also been implicated in tolerance to cold stress. *VaBAM1* from grape (*Vitis amurensis*) is activated by cold stress, and the overexpression of *VaBAM1* promotes freezing tolerance in Arabidopsis [23]. In rice, suppressing *BAM* genes leads to decreased cold tolerance [24]. *PtrBAM1* from *Poncirus trifoliata* was induced by cold, and the overexpression of it in tobacco increased sugar content and conferred greater cold tolerance [25]. Furthermore, *PbrBAM3* regulates soluble sugar accumulation and ROS homeostasis in pear (*Pyrus betulaefolia*) under cold stress [13]. All these studies demonstrate that *BAM* genes are induced by stress factors and highlight the essential role of BAM in tolerance to cold stress in plants. However, the mechanism underlying the induction of *BAMs* under stress conditions remains largely unknown, especially under cold conditions. It is also elusive regarding the upstream regulators of *BAM* genes.

Jasmonates (JAs), a class of lipid-derived plant hormones, were first identified as defense hormones protecting plants against biotic stresses. A growing body of evidence supports that JA is also a key player in many aspects of growth, development, and abiotic stress responses. Previous investigations have pointed out that JA regulates root development, leaf senescence, seed size, heat tolerance, and drought tolerance, among others [26,27,28,29]. In the past decade, emerging evidence has demonstrated that JA acts as a vital hormone involved in the cold response [30,31]. Direct evidence arises from observations that, during cold stress, the accumulation of JA is enhanced in Arabidopsis, tomato, apple, *Artemisia annua*, etc. [32,33,34,35]. In addition, the exogenous feeding of MeJA strengthens cold tolerance in banana, orange, apple, tomato, etc. [36,37,38]. JA enhances cold tolerance by degrading JAZs and activating the ICE-CBF module, a conserved module important for cold responses across species. Similarly, JA promotes tomato tolerance to cold stress through the MYC2-activiated glutathione *S*-transferase gene *SlGSTU24* [38]. Additionally, JA acts in concert with melatonin and ethylene to alleviate cold stress-induced damage in tomato plants [39]. Interestingly, under cold conditions, JA triggers the biosynthesis of betaine, a key osmolyte required for efficiently adapting to or tolerating stress [9]. Yet, the impact of JA on the accumulation of soluble sugars in response to cold stress remains largely unresolved. Importantly, given that BAM-mediated starch degradation is essential for the production of soluble sugars, the regulatory relationship between JA signaling and *BAM* expression under cold stress remains confusing.

Tomato (*Solanum lycopersicum*) is arguably one of the most popular vegetable crops across the globe because of its well-recognized nutritional value. Tomatoes are important sources of nutrients, such as vitamin C, vitamin E, folate, trace elements, flavonoids, lycopene, and potassium, which are beneficial to health [40,41]. Though tomatoes are grown and consumed worldwide, they originated in warm regions in South America, rendering the species susceptible to chilling and freezing conditions. Low temperatures are important environmental factors that severely restrict growth, development, yield, and quality in tomatoes, threatening the sustainable development of the tomato industry [42,43]. It is thus crucial to determine the molecular and physiological mechanisms of tomato’s cold response.

In this work, we report a tomato BAM gene, *SlBAM3*, that acts positively in tomato tolerance to cold stress by promoting starch degradation, increasing sugar accumulation, and strengthening ROS detoxification. We found that MYC2, which is a core mediator of JA signaling, directly activated *SlBAM3* transcription and positively regulated cold tolerance. Together, these results support the hypothesis that JA positively regulates β-amylase-associated starch degradation through a JA-dependent module, MYC2-SlBAM3, in tomato during cold stress. This study enhances our comprehension of the underlying mechanisms that trigger *BAM* gene activation and starch catabolism under cold stress. Additionally, our work illuminates the connection between JA and the *BAM* genes in tomato under cold stress. The MYC2-SlBAM3 module can be further utilized to breed tomato cultivars with enhanced cold tolerance.

## 2. Results

### 2.1. Cold Stress Enhances SlBAM3 Expression, Accelerates Starch Degradation, and Promotes Soluble Sugar Accumulation in Tomato Leaves

**Figure 1:**
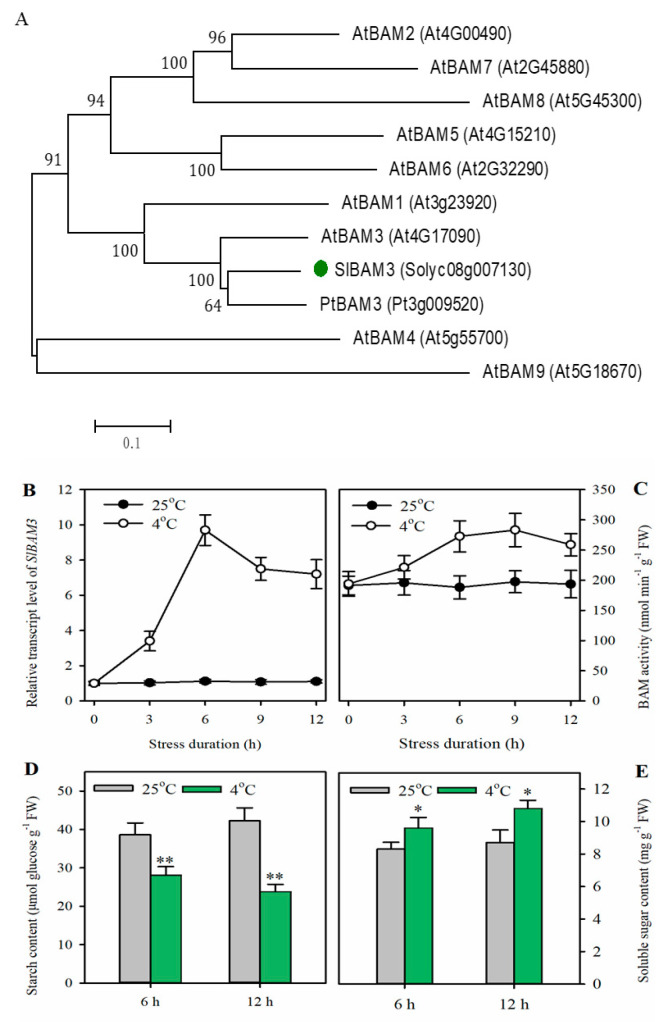
Cold stress induces *SlBAM3* expression, starch degradation, and soluble sugar accumulation in tomato leaves. (**A**) Phylogenetic tree of *SlBAM3*, *PtBAM3*, and Arabidopsis *BAMs*. Tree construction was carried out using MEGA 6.06. A consensus neighbor−joining tree was obtained from 1000 bootstrap replicates of aligned sequences. (**B**) Time course of the *SlBAM3* transcript level under cold stress (4 °C). (**C**) Time course of β-amylase activity under cold stress. (**D**) Starch content. (**E**) Soluble sugar content. The values shown in this figure are means ± SDs (*n* = 3). Student’s *t*-test was used to analyze difference between samples under cold stress and those under normal growth conditions (* *p* < 0.05 or ** *p* < 0.01).

To understand how low temperatures affect starch catabolism and sugar production, we first aimed to identify the tomato *BAM* genes responsible for leaf starch degradation under cold stress. Through an analysis of RNA-Seq data (NCBI SRA accession no. SRR18694252), we identified four *BAM* genes (Solyc08g007130, Solyc09g091030, Solyc07g052695, Solyc07g052690) that were significantly changed in FPKM (FC > 2) after cold treatment. A *BAM* gene (Solyc08g007130) with a tremendous increase in FPKM (FC > 80) was selected for further investigation. We performed a phylogenetic analysis of this gene, *PtrBAM3* from *Poncirus trifoliata*, and nine *BAM* genes from Arabidopsis, and found that the tomato *BAM* gene has the closest evolutionary relationships with *PtrBAM3* and *AtBAM3* (Figure 1A). We named the identified tomato gene *SlBAM3*. Next, we examined the time course of *SlBAM3* transcript levels after cold treatment (4 °C) under light. The expression of *SlBAM3* was rapidly induced by cold and the highest transcript level was observed at 6 h (Figure 1B). Consistently, cold-treated tomato plants exhibited markedly higher β-amylase activity than the control plants. β-amylase activity was increased by 43% in cold-treated plants compared to control plants at 9 h after cold treatment (Figure 1C). Since cold stress significantly induced the expression of *SlBAM3*, we expected an obvious decrease in starch content. To determine starch content, tomato plants were first allowed to grow under light at 25 °C for 12 h before they were treated under light at 4 °C. It was shown that cold stress reduced starch accumulation in tomato leaves by 27% and 44% at 6 h and 12 h, respectively, after cold treatment (Figure 1D). Contrary to the starch content, the soluble sugar content was increased in tomato leaves during cold treatment. The levels of soluble sugars were elevated by 16% and 24% at 6 h and 12 h, respectively, during cold treatment (Figure 1E). These results imply that *SlBAM3* is a β-amylase gene associated with starch degradation and the accumulation of soluble sugars in tomato leaves subjected to cold stress.

### 2.2. Knockout of SlBAM3 Impairs Cold Tolerance in Tomato Leaves

**Figure 2:**
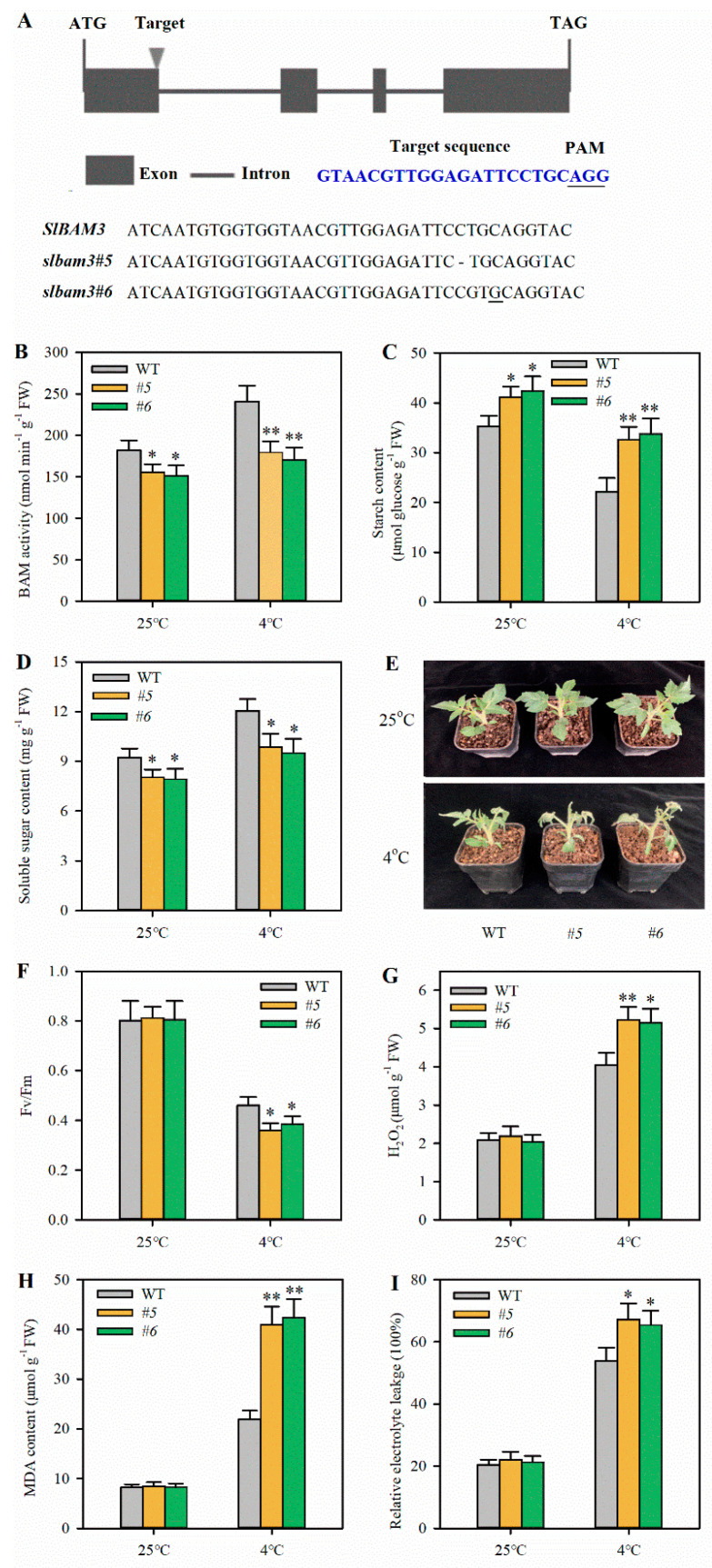
Knockout of *SlBAM3* decreases cold tolerance in tomato. (**A**) Schematic diagram of the target region of *SlBAM3* and two mutant alleles in T_2_ plants. (**B**) β-amylase activity. (**C**) Starch content. (**D**) Soluble sugar content. (**E**) Phenotypes of mutant lines before and after cold stress. (**F**) Fv/Fm. (**G**) H_2_O_2_ content. (**H**) MDA content. (**I**) REL. The values shown in this figure are means ± SDs (*n* = 3). For Fv/Fm, *n* = 15. The values shown in this figure are means ± SDs (*n* = 3). Student’s *t*-test was used to analyze differences between mutant plants and wild-type plants either under normal growth conditions or cold stress (* *p* < 0.05 or ** *p* < 0.01).

We observed that *SlBAM3* was up-regulated by cold stress, concurrent with the decrease in starch content and the increase in soluble sugar content. These observations led us to speculate that *SlBAM3* has a positive role in tomato cold tolerance. To verify this speculation, we obtained *SlBAM3* knockout mutants using a CRISPR/Cas9 gene-editing system and used them to evaluate starch catabolism and cold tolerance (Figure 2A). Two transgenic lines carrying a 1 bp deletion and a 1 bp insertion in the first exon of *SlBAM3* were selected. Both mutations resulted in an early stop codon and produced severely truncated protein that was predicted to be without a catalytic domain. T2 plants with homozygous mutations were selected for further study. An enzyme activity assay showed that *slbam3* mutants displayed prominently lower β-amylase activities than wild-type plants, in particular, under cold stress (Figure 2B). In parallel, mutant lines had more starch than the control plants under cold conditions (Figure 2C). On the contrary, the accumulation of soluble sugars was markedly lower in *slbam3* mutant plants than that in the wild-type ones under cold stress (Figure 2D). Both mutant and wild-type plants showed no phenotypic differences under a normal growth temperature; however, *slbam3* mutants displayed more severe damage than wild-type plants under cold stress (Figure 2E). Next, we measured Fv/Fm, hydrogen peroxide (H_2_O_2_) content, relative electrolyte leakage (REL), and malondialdehyde (MDA) content to assess cold tolerance. *slbam3* mutants showed significantly decreased Fv/Fm in comparison with the control plants after cold treatment, although both genotypes of plants had similar Fv/Fm under normal growth conditions (Figure 2F). In addition, the mutant lines exhibited remarkably higher levels of H_2_O_2_ and MDA, and substantially more REL, relative to the wild-type control under cold conditions (Figure 2G–I). These observations imply that *SlBAM3* positively modulates cold tolerance by promoting starch degradation and soluble sugar accumulation in tomato.

### 2.3. JA Induces SlBAM3 Expression and Enhances BAM Activity under Cold Stress

**Figure 3:**
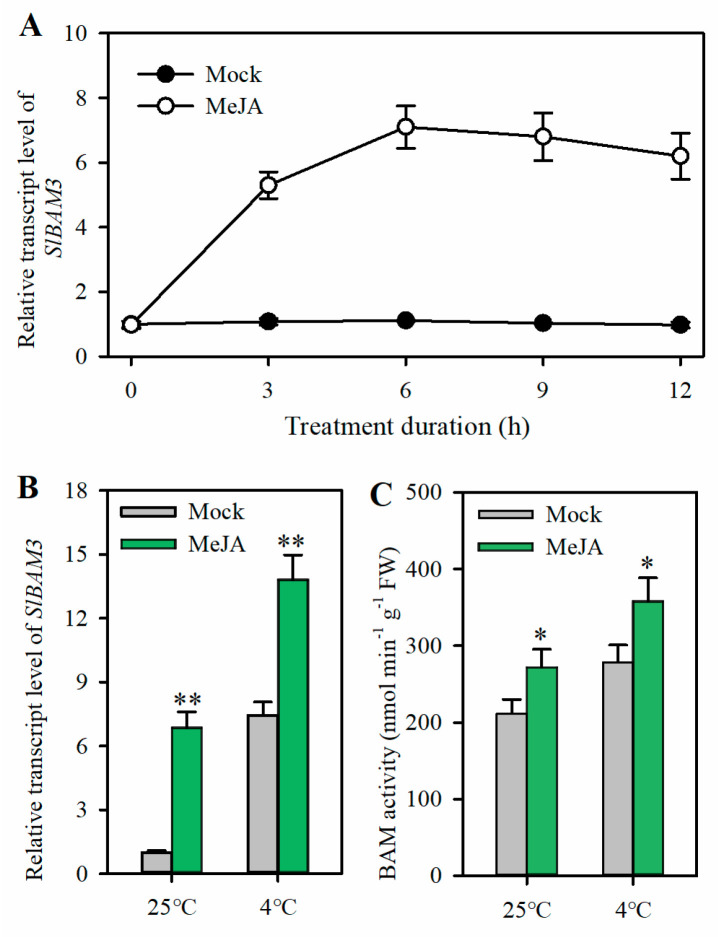
MeJA induces the expression of *SlBAM3* and stimulates β-amylase activity. (**A**) Time course of the *SlBAM3* transcript levels in response to MeJA. (**B**) *SlBAM3* transcript level in MeJA−treated tomato leaves under cold stress. (**C**) β-amylase activity in MeJA-treated tomato leaves under cold stress. The values shown in this figure are means ± SDs (*n* = 3). Student’s *t*-test was used to analyze differences between MeJA and mock treatments either under normal growth conditions or cold stress (* *p* < 0.05 or ** *p* < 0.01).

JA has been demonstrated to function positively in the cold response in plants. In this study, it was observed that *SlBAM3* enhances cold tolerance in tomato. However, whether JA associates with *SlBAM3* in cold response remains enigmatic. To uncover the relationship between them, we first analyzed the RNA-seq data (PRJCA000395, NGDC) of MeJA-treated tomato seedlings. We found that *SlBAM3* transcripts were significantly increased by MeJA treatment. Subsequent qRT-PCR analysis confirmed the RNA-seq results. Time-course analysis revealed that the relative expression of *SlBAM3* was tremendously elevated 3 h after MeJA treatment (Figure 3A). Next, we tested the role of MeJA in the induction of *SlBAM3* under cold stress. Detached tomato leaves were first floated in MeJA solution and incubated under light at 25 °C for 12 h; then, they were placed under light at 4 °C. The level of *SlBAM3* transcript in tomato leaves treated with MeJA was significantly higher than that in leaves treated with mock solution, and the difference was particularly prominent after a 12 h of cold treatment (Figure 3B). In agreement with the level of *SlBAM3* transcript, β-amylase activity was obviously enhanced by MeJA under cold stress (Figure 3C). These results indicate that JA triggers *SlBAM3* expression and stimulates β-amylase activity under cold stress.

### 2.4. MYC2 Directly Activates the Expression of SlBAM3

**Figure 4:**
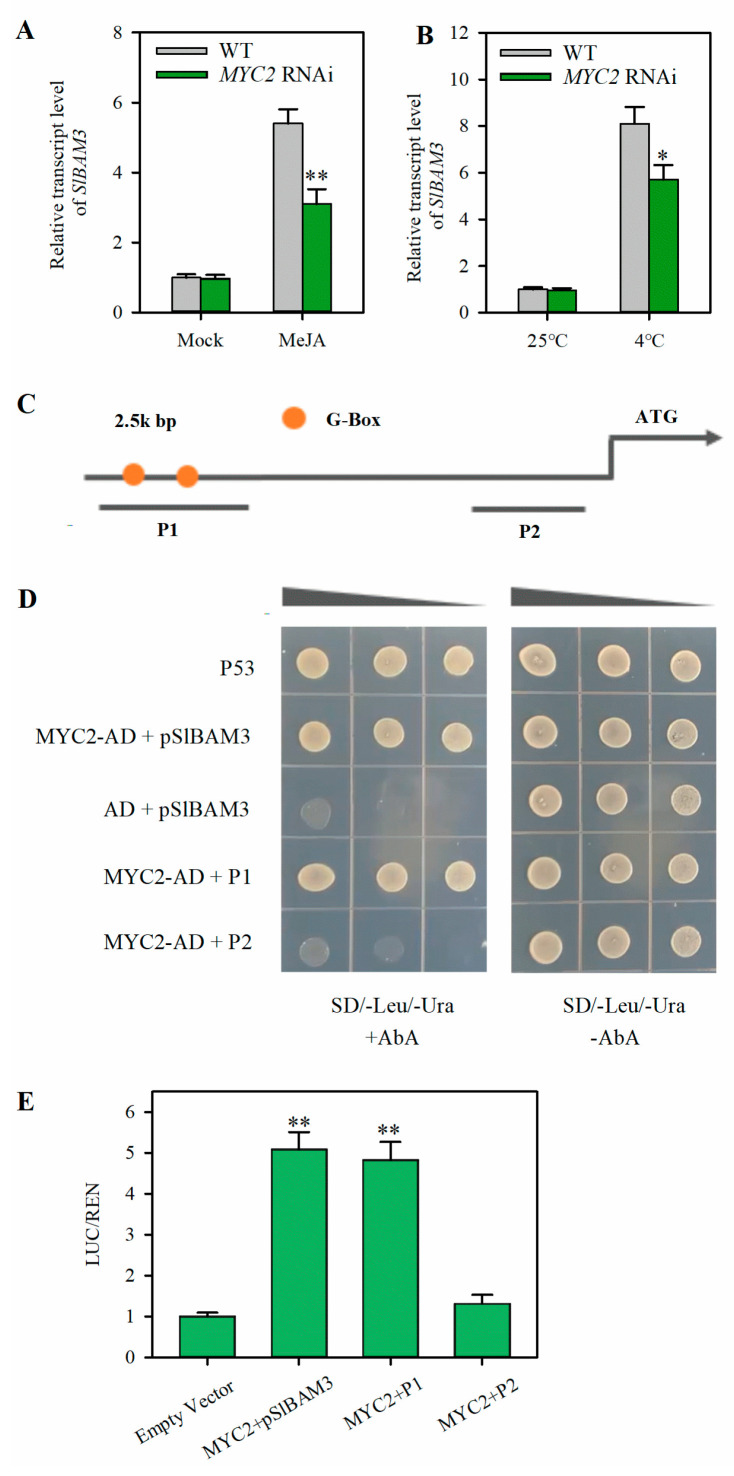
MYC2 directly regulates the expression of *SlBAM3.* (**A**) *SlBAM3* transcript level in *MYC2* RNAi plants following MeJA treatment. The values shown are means ± SDs (*n* = 3). Student’s *t*-test was used to analyze differences between MeJA and mock treatments either under normal growth conditions or cold stress (* *p* < 0.05 or ** *p* < 0.01). (**B**) *SlBAM3* transcript level in *MYC2* RNAi plants following cold treatment. The values shown are means ± SDs (*n* = 3). (**C**) Schematic representation of the *SlBAM3* promoter. The circles indicate the predicted binding sites of MYC2. (**D**) Y1H analysis of MYC2 binding to the *SlBAM3* promoter. (**E**) LUC/REN analysis of the role of MYC2 in *SlBAM3* promoter activity. The values shown are means ± SDs (*n* = 3). Student’s *t*-test was used to analyze differences between MYC2−*pSlBAM3*, MYC2−*P1*, or *MYC2*−*P2* and empty vector (** *p* < 0.01).

Although JA stimulates the expression of *SlBAM3*, the underlying mechanism has yet to be explored. As MYC2 is a core mediator of JA signaling, next, we asked whether the JA-induced increase in *SlBAM3* transcript is relevant to MYC2. We first examined the transcript levels of *SlBAM3* in *MYC2* RNAi lines that we had previously generated [28]. After MeJA treatment, the expression of *SlBAM3* was considerably increased; however, the increase in the *SlBAM3* transcript level was abated in *MYC2* RNAi plants (Figure 4A). Under normal a growth temperature, only a slight difference in the relative expression of *SlBAM3* was observed between *MYC2* RNAi lines and wild-type plants; however, under cold conditions, a substantially lower level of *SlBAM3* transcript was detected in *MYC2* RNAi lines than that in wild-type plants (Figure 4B), suggesting a positive role of MYC2 in the regulation of *SlBAM3* under cold conditions. Next, we considered it reasonable to investigate whether MYC2 directly regulates *SlBAM3*. To answer this question, we performed an analysis of the promoter of *SlBAM3* using the webtool PlantCARE. Two binding sites (G-box) were present in the *SlBAM3* promoter (Figure 4C). It is well established that MYC2 shows preference for G-box in the promoter region, suggesting that MYC2 may associate with the *SlBAM3* promoter. To verify this association, a Y1H assay was performed. It was shown that yeast cells with both MYC2 and the full-length promoter (2500 bp) were able to grow in a selection medium supplemented with Aureobasidin A (AbA). As expected, yeast cells harboring MYC2 and a promoter fragment (P1) with two G-box sites also grew in the selection medium. However, the replacement of P1 with a fragment without G-box (P2) completely abolished the ability to grow in the selection medium (Figure 4D). The results of the Y1H assay indicate that MYC2 binds directly to the *SlBAM3* promoter through the *cis*-acting elements of G-box. To further substantiate the role of MYC2 in the activation of *SlBAM3*, we proceeded to conduct a dual LUC assay. The effector construct *35S:SlMYC2* was co-expressed either with the reporter construct *pSlBAM3:LUC* (full-length promoter), *P1:LUC* (promoter fragment with predicted MYC2 binding site), or *P2:LUC* (promoter fragment without predicted MYC2 binding site) in tobacco leaves. The relative LUC activity as represented by the LUC/REN ratio was markedly enhanced when tobacco (*N. benthamiana*) leaves were inoculated with *A. tumefaciens* harboring MYC2 and the full-length promoter. However, when the full-length promoter sequence was replaced with a promoter fragment without G-box, the relative LUC activity was decreased to the control level (Figure 4E). These observations indicate that MYC2 transcriptionally activates *SlBAM3* by binding directly to G-box in the *SlBAM3* promoter.

### 2.5. Suppression of MYC2 Reduces Soluble Sugar Accumulation and Attenuates Cold Tolerance

**Figure 5:**
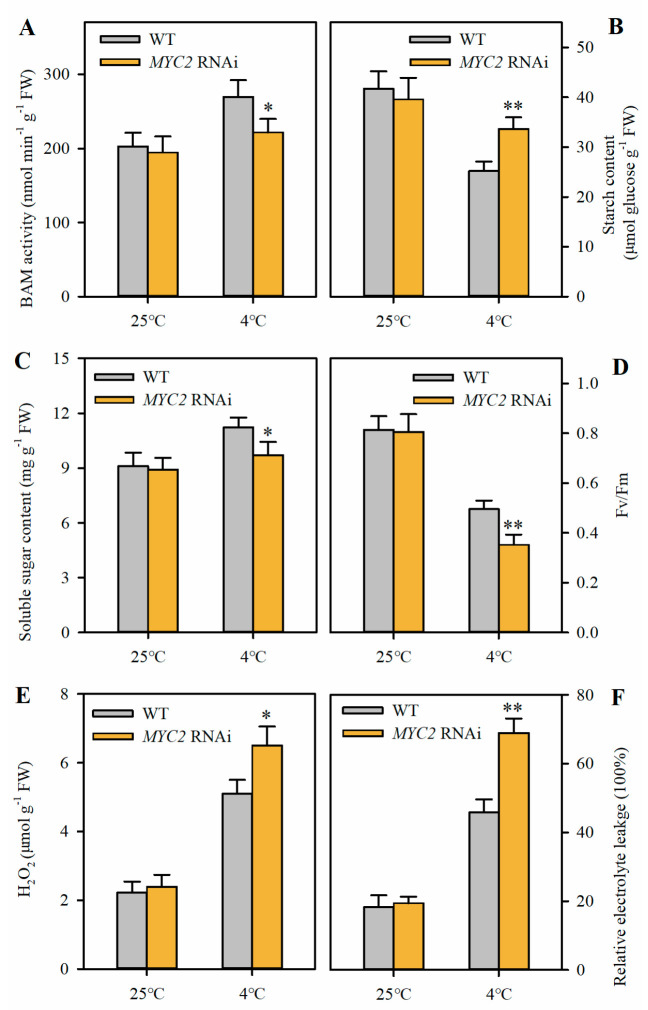
Suppression of *MYC2* reduces β-amylase activity and decreases cold tolerance. (**A**) β-amylase activity. (**B**) Starch content. (**C**) Soluble sugar content. (**D**) Fv/Fm. (**E**) H_2_O_2_ content. (**F**) REL. The values shown in this figure are means ± SDs (*n* = 3). For Fv/Fm, *n* = 15. Student’s *t*-test was used to analyze differences between *MYC2* RNAi plants and wild-type plants (* *p* < 0.05 or ** *p* < 0.01).

Having observed that MYC2 directly targets *SlBAM3* and regulates its transcription, we next examined the role of MYC2 in starch catabolism and cold tolerance in tomato leaves. An enzyme activity assay showed that the *MYC2* RNAi lines and the control plants displayed no significant differences in BAM activity under a normal growth temperature; however, the *MYC2* RNAi lines exhibited significantly lower BAM activity than the control plants under cold stress (Figure 5A). Concurrently, the *MYC2* RNAi lines accumulated considerably more starch but less soluble sugars than their wild-type counterparts after cold treatment (Figure 5B,C). Not surprisingly, Fv/Fm was significantly decreased in the *MYC2* RNAi lines relative to Fv/Fm in the control plants under cold stress (Figure 5D). In addition, both H_2_O_2_ content and REL were tremendously increased in the *MYC2* RNAi lines compared to those in the control plants after cold treatment (Figure 5E,F). These results imply that MYC2 functions positively in cold tolerance, at least in part, dependent on *SlBAM3*-mediated starch degradation and soluble sugar accumulation.

## 3. Discussion

**Figure 6:**
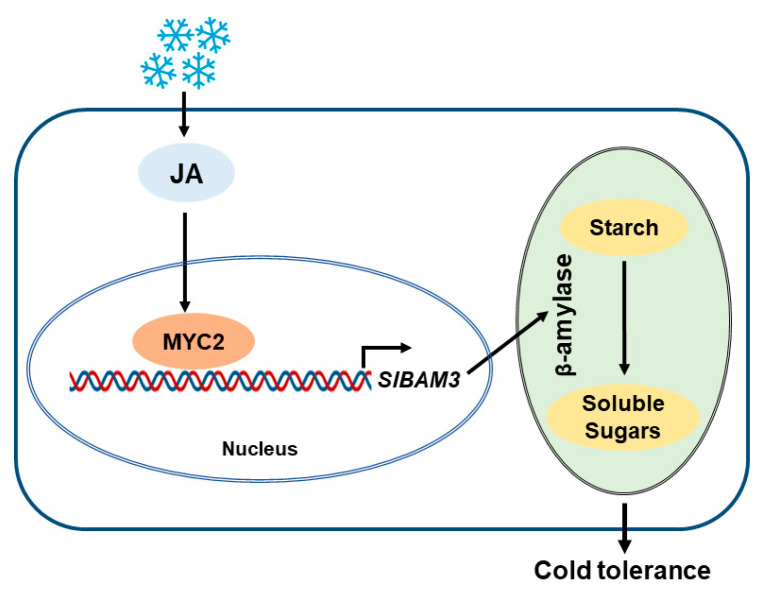
A proposed regulatory pathway for *SlBAM3*-mediated starch degradation in tomato plants under cold conditions. Under cold stress, JA enhances *SlBAM3* expression through MYC2, which directly activates *SlBAM3* transcription, thus promoting starch degradation and soluble sugar accumulation, ultimately leading to increased tolerance in tomato plants.

Cold is a critical environmental factor that severely threatens crop growth, development, and yields. The sessile nature of plants has led to the evolution of an intricate defense network that enables them to survive under low-temperature stress. The accumulation of soluble sugars is one crucial strategy to cope with cold stress, as soluble sugars are capable of maintaining membrane integrity, stabilizing proteins, and scavenging excessive ROS. During stress, the accumulation of soluble sugar is largely dependent on starch degradation, a process controlled by multiple enzymes, among which β-amylase (BAM) is of great importance. Tomato is a vegetable crop with great popularity; however, tomato production is often restricted by cold stress. So far, it remains largely obscure with respect to BAM-mediated starch degradation in tomato plants under cold stress. In particular, the upstream factors that regulate the expression of *BAM* genes are poorly unraveled in tomatoes. In this work, we report a key tomato BAM gene, *SlBAM3*, which is of vital importance for starch degradation, soluble sugar accumulation, and cold tolerance. Importantly, we identified MYC2 as a key transcription factor that activates *SlBAM3*. MYC2 is a critical regulator in the JA signaling pathway, thus highlighting the role of JA in the regulation of *SlBAM3*-mediated starch degradation and cold tolerance in tomato (Figure 6).

Starch catabolism in plants is regulated by the internal circadian clock and external environmental clues. Previous studies have revealed that starch degradation is accelerated by environmental factors, including drought, high/low temperatures, salinity, and blue light [13,20,21,23,44]. In this study, starch level was drastically reduced upon cold treatment in tomato, and we provide several lines of evidence supporting that *SlBAM3* plays a major role in starch degradation and soluble sugar accumulation in tomato during cold stress. Firstly, *SlBAM3* was remarkably induced, accompanied by enhanced BAM activities in tomato leaves, under cold conditions. Second, the accumulation of starch was decreased, while soluble sugar content was increased, in tomato leaves after cold treatment. Third, the knockout mutation of *SlBAM3* resulted in a higher accumulation of starch and a lower soluble sugar content under cold stress. Finally, the mutation of *SlBAM3* attenuated cold tolerance, as revealed by various physiological assays. Collectively, *SlBAM3* is a crucial player in starch degradation, soluble sugar accumulation, and cold tolerance in tomato. 

In most cases, starch degradation is enhanced by abiotic stress. For instance, in *P. trifoliata*, starch degradation was promoted in response to dehydration [19]. In woody lychee trees and herbaceous barley, starch was degraded upon osmotic stress [45,46], and in our study, starch degradation was accelerated in tomato leaves after cold treatment. However, several studies have reported conflicting results. An increase in starch accumulation was observed in *Thellungiella halophila*, *Chlamydomonas reinhardtii*, and Arabidopsis under stressful conditions [47,48,49]. Contrary to what was observed in tomato leaves after cold treatment in our study, it is puzzling that cold stress resulted in an increase in starch content in tomato leaves in a previous study [50]. This difference may arise from the type of stress and the experimental conditions. Importantly, these conflicting observations emphasize the complex regulation of starch metabolism under stress conditions. Another difference between our results and previous results that should be noted is the change in BAM activity under cold conditions. In our study, in line with the increased levels of *SlBAM3* transcript, BAM activity was enhanced under cold stress. However, in an earlier study, although *BAM3* transcript levels were strongly induced, BAM activity was significantly inhibited under cold stress [51]. This discrepancy may be due to different sampling times after cold treatment, as prolonged cold stress may compromise the catalytic capability of BAM. The difference may also be due to the species specificity of BAMs. These contradictory results of different studies warrant further investigation.

The transcription of *BAM* genes in response to various stresses is, to a large extent, determined by transcription factors involved in various signaling pathways. For example, PtrABF4 and PtrABR1 function as direct activators of *PtrBAM3* in *P. trifoliata* under dehydration conditions [19]. Moreover, as a key regulator in the light signaling pathway, HY5 plays a vital role in the blue-light-induced starch degradation by promoting the expression of starch catabolic genes in tomato, including *BAM1*, *BAM3*, and *BAM8* [44]. In our study, we revealed that JA-induced *SlBAM3* expression is achieved, at least partially, through MYC2, a crucial mediator of JA signaling. This conclusion is supported by several results. First of all, the down-regulation of *MYC2* reduced the level of *SlBAM3* transcripts in tomato leaves under cold conditions. Second, MYC2 could activate *SlBAM3* transcription through direct binding to its promoter, as demonstrated by the Y1H and dual LUC assays. Lastly, the suppression of *MYC2* attenuated *SlBAM3*-mediated starch degradation and cold tolerance in tomato plants. Together, these pieces of evidence favor JA in promoting starch degradation through MYC2, which activates the transcription of *SlBAM3* in tomato leaves under cold stress. Interestingly, an earlier study demonstrated that herbivory-induced jasmonates reduced sugar accumulation in plants [52], which is distinct from the JA-induced starch degradation and soluble sugar accumulation observed in our study. It is possible that plants utilize JA signaling to promote or decrease sugar accumulation depending on the type of stress (biotic or abiotic) they encounter. More studies are needed to fully explain the difference.

Based on all observations in this study, we proposed a regulatory pathway for *SlBAM3*-mediated starch degradation in tomato plants under cold conditions. Basically, JA enhances *SlBAM3* expression through MYC2, which directly activates *SlBAM3* transcription, thus promoting starch degradation and soluble sugar accumulation, ultimately leading to increased tolerance in tomato plants. The discovery of the MYC2-SlBAM3 module improves our understanding of the mechanisms underlying starch metabolism in tomato in response to abiotic stress. This study also provides valuable gene resources for the breeding of vegetable crops with improved cold tolerance.

## 4. Materials and Methods

### 4.1. Plant Materials

In this study, various genotypes of tomato plants were used as plant materials, including wild-type plants (*Solanum lycopersicum* ‘MicroTom’), *slbam3* mutant lines, and *MYC2* RNAi lines. The wild-type and mutant plants were maintained under a long-day photoperiod condition (16 h/8 h) in growth chambers, with the light intensity measured at a photosynthetic photon flux density (PPFD) of 300 µmol m^−2^ s^−1^. The air temperature followed a diurnal pattern, at 25 °C during the day and 22 °C at night. The relative air humidity inside the chambers was approximately 65%.

### 4.2. Treatments

At the 4-leaf stage, unless otherwise stated, tomato plants were used for different treatments and assessments. For cold treatment, tomato plants were first grown at 25 °C under light for 12 h, and then, subjected to 4 °C under light for 24 h. Fully expanded young leaves were collected at the designated time for physiological and phenotypic analysis. For the treatments with JA, fully expanded young leaves at the 4-leaf stage were detached and floated in JA solution (10 µM) for 12 h to determine the level of *SlBAM3* transcript. To examine the role of JA in the induction of *SlBAM3* expression and β-amylase activity, detached tomato leaves were first treated with JA before these leaves were treated at a low temperature (4 °C) under light for 12 h.

### 4.3. Cold Tolerance Assay

To evaluate the tolerance of tomato plants to cold stress, we assessed various indicators, including Fv/Fm, relative electrolyte leakage (REL), malondialdehyde (MDA) content, and hydrogen peroxide (H_2_O_2_) level. The Fv/Fm value was determined using a chlorophyll fluorometer, following the methodology reported by Wang and colleagues [7]. Briefly, the minimal fluorescence (Fo) was measured from dark-adapted plants using a portable fluorometer (PAM-2000, Walz, Effeltrich, Germany), and the maximal fluorescence from a dark-adapted leaf (Fm) was obtained following a saturating pulse, allowing us to calculate the maximum quantum efficiency (Fv/Fm). The measurement of REL was conducted according to the protocol described by Ding et al. [39]. MDA content was measured according to a published work [7]. H_2_O_2_ was quantified according to an earlier study [4].

### 4.4. CRISPR/Cas9-Mediated Mutation of SlBAM3

Knockout mutants of *SlBAM3* were generated using a CRISPR/Cas9 gene editing tool. The target sequence (GTAACGTTGGAGATTCCTGCAGG) was selected using a webtool of CRISPR-P [53]. The target fragment was inserted into the vector AtU6-sgRNA-AtUBQ-Cas9. The resulting vector was then ligated to the binary vector pCAMBIA1301. After that, the reconstructed vector was transformed into *A. tumefaciens* (GV3101), which was later employed for plant transformation according to an earlier report [43]. The T_2_ homozygous plants were used for the following experiments. 

### 4.5. Determination of Carbohydrate Accumulation and Enzyme Activity

The fully expanded young leaves after different treatments were used to determine the starch content, soluble sugar content, and β-amylase activity. Starch accumulation was measured using a commercial kit (Solarbio, Beijing, China). The soluble sugar content was determined as previously described [50]. β-amylase activity was assayed as described in an earlier study [54].

### 4.6. Yeast One-Hybrid (Y1H) Assay

To investigate the binding of MYC2 to the *SlBAM3* promoter, Y1H was carried out. Briefly, different fragments of the *SlBAM3* promoter were cloned into the pAbAi vector, while the complete coding sequence of MYC2 was linked to the pGADT7 vector. Subsequently, both modified vectors were introduced into the Y1H Gold yeast strain. The yeast cells were cultured under two conditions: SD/-Ura/-Leu medium, with or without the addition of 100 ng mL^−1^ AbAi, at a temperature of 30 °C for a duration of 3 days. The association of MYC2 with the *SlBAM3* promoter was determined by assessing the growth capacity of transformed yeast cells on the selection medium.

### 4.7. Gene Expression Analysis

To examine the relative expression of each gene, qRT-PCR was performed as reported in a previous study [28]. Concisely, total RNA was isolated using an extraction kit (RNAprep Pure Plant Kit, TIANGEN, Beijing, China) and subsequently used as a template to synthesize cDNA. Finally, the qRT-PCR reaction was conducted on a real-time PCR system with a commercial kit (Premix Ex Taq kit, TaKaRa, San Jose, CA, USA). *SlACTIN2* was used as an internal reference. The primers used for qRT-PCR analysis can be found in Supplementary Figure S1.

### 4.8. Dual Luciferase Assay

The vectors pGreenII 62-SK and pGreenII0800-LUC were used for the dual luciferase assay according to an earlier report [38]. The CD of *MYC2* was ligated to the vector pGreenII 62-SK and the resulting recombinant vector served as the effector. The promoter fragments of *SlBAM3* with or without G-Box were inserted into pGreenII0800-LUC and the reconstructed vector was used as the reporter. Subsequently, the effector and reporter constructs were transformed into *A. tumefaciens* (GV3101), which was then used for transient expression in *N. benthamiana* leaves. LUC activities were determined using a commercial assay kit (Promega, Madison, WI, USA).

### 4.9. Statistical Analysis

In this study, the differences between the groups were compared using Student’s *t*-test. Significant differences are designated by asterisks (* *p* < 0.05 and ** *p* < 0.01). All experiments in this study were performed with at least three biological replicates.

## Data Availability

The data are contained within the article.

## Supplementary Materials

The following supporting information can be downloaded at: https://www.mdpi.com/article/10.3390/plants13081055/s1, Figure S1: Relative expression of SlBAM3 correlates with BAM activity. Data used for correlation analysis were from Figure 1 and Figure 5.
