# Supplementary material for: Jasmonates Promote β-Amylase-Mediated Starch Degradation to Confer Cold Tolerance in Tomato Plants
Source: Plants (Basel). 2024 Apr 9;13(8):1055. doi: 10.3390/plants13081055 (PMC11055051; doi:10.3390/plants13081055)
Supplement: Supplementary file 1 [file plants-13-01055-s001.zip › plants-2711380-supplementary.pdf]

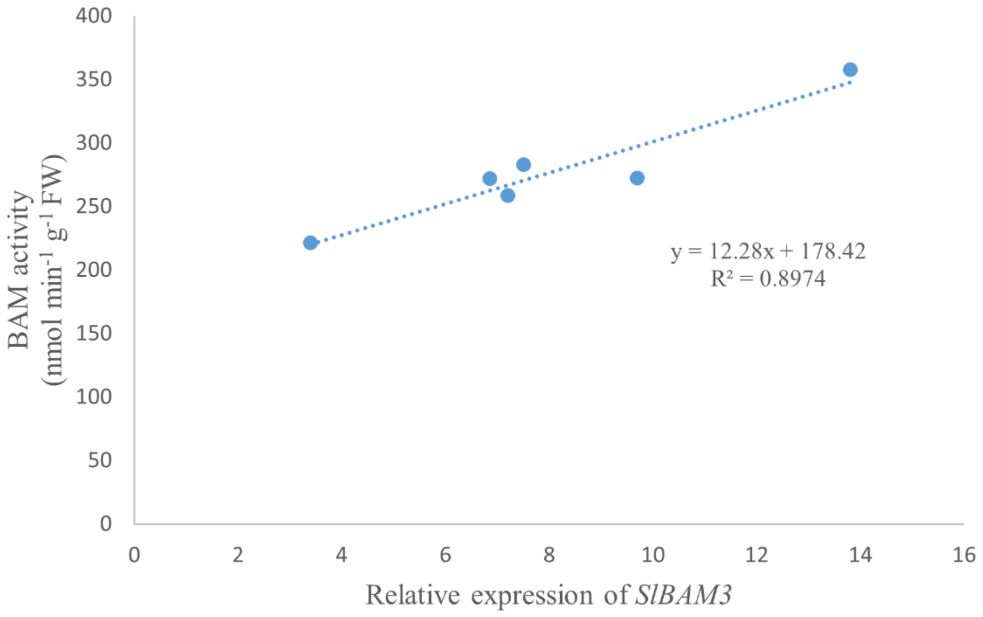

Supplementary Figure S1. Relative expression of *SlBAM3* correlates with BAM activity. Data used for correlation analysis were from Figures 1 and 5.
